# Absolute quantitation of human wild-type DNAI1 protein in lung tissue using a nanoLC-PRM-MS-based targeted proteomics approach coupled with immunoprecipitation

**DOI:** 10.1186/s12014-024-09453-0

**Published:** 2024-02-04

**Authors:** Hui Wang, Xiaoyan Ni, Nicholas Clark, Kristen Randall, Lianne Boeglin, Sudha Chivukula, Caroline Woo, Frank DeRosa, Gang Sun

**Affiliations:** 1 Translate Bio, a Sanofi Company, Lexington, MA 02421 USA; 2 Sanofi, Waltham, MA 02451 USA

**Keywords:** Human DNAI1, PCD, Protein quantitation, Targeted proteomics, Immunoprecipitation, PRM, Mass spectrometry, Tissue

## Abstract

**Background:**

Dynein axonemal intermediate chain 1 protein (DNAI1) plays an essential role in cilia structure and function, while its mutations lead to primary ciliary dyskinesia (PCD). Accurate quantitation of DNAI1 in lung tissue is crucial for comprehensive understanding of its involvement in PCD, as well as for developing the potential PCD therapies. However, the current protein quantitation method is not sensitive enough to detect the endogenous level of DNAI1 in complex biological matrix such as lung tissue.

**Methods:**

In this study, a quantitative method combining immunoprecipitation with nanoLC-MS/MS was developed to measure the expression level of human wild-type (WT) DNAI1 protein in lung tissue. To our understanding, it is the first immunoprecipitation (IP)-MS based method for absolute quantitation of DNAI1 protein in lung tissue. The DNAI1 quantitation was achieved through constructing a standard curve with recombinant human WT DNAI1 protein spiked into lung tissue matrix.

**Results:**

This method was qualified with high sensitivity and accuracy. The lower limit of quantitation of human DNAI1 was 4 pg/mg tissue. This assay was successfully applied to determine the endogenous level of WT DNAI1 in human lung tissue.

**Conclusions:**

The results clearly demonstrate that the developed assay can accurately quantitate low-abundance WT DNAI1 protein in human lung tissue with high sensitivity, indicating its high potential use in the drug development for DNAI1 mutation-caused PCD therapy.

**Supplementary Information:**

The online version contains supplementary material available at 10.1186/s12014-024-09453-0.

## Introduction

Primary ciliary dyskinesia (PCD) is a rare genetic disorder characterized by dysfunctional and structurally abnormal ciliary. The disease has a serious impact on the respiratory system including the sinuses, ears, and lungs, leading to repeated respiratory infections, pneumonia, as well as infertility. In severe cases, patients with PCD often develop respiratory failure and even death. The estimated prevalence of PCD is around one in 10,000–20,000 live births [1–5]. So far, there is no curative treatment for PCD. Current treatments are limited to prevent and manage the disease complications [6–8]. Therefore, new therapeutic approaches are required to address this urgent unmet need.

Among more than 40 known PCD-causative genes, mutations on Dynein axonemal intermediate chain 1 (DNAI1) gene have been recognized as one of the most important genetic causes and account for approximately 10–14% of the cases in PCD [9–14]. In recent years, gene therapy, especially mRNA-based therapeutics, has been proposed as a prospective treatment for PCD [15–18]. In vitro and in vivo data of promising gene therapeutics demonstrated this great potential [8]. Most recently, ReCode Therapeutics, a genetic medicines company, announced the initiation of Phase 1 clinical trial of RCT1100, a first-in-class, mRNA-based genetic medicine for the treatment of people with PCD caused by pathogenic mutations in the DNAI1 gene, and first participants have been dosed to evaluate safety and tolerability of RCT1100 (https://recodetx.com/).

Given the vital role of DNAI1 in PCD, accurate quantitation of DNAI1 expression in vivo is very crucial for understanding PCD pathophysiology and mechanistic cellular processes, as well as for developing potential therapeutic interventions. Quantitation of DNAI1 also provides important information to evaluate the potency of mRNA-based therapeutics and can be used as a in vivo biomarker in pre-clinic and clinic PK/PD studies in the drug development of mRNA-based genetic medicines. However, due to the low endogenous level of DNAI1 protein, the quantitation of DNAI1 in complex biological samples such as lung tissue is extremely challenging. So far, commercially available ELISA or western blot kits specific designed for DNAI1 protein analysis are limited to DNAI1 detection in cell lysate and isolated ciliary axonemes [19, 20].

As a robust and highly sensitive tool, mass spectrometry (MS) has been widely used in low-abundance protein quantitation. However, only a small number of publications reported MS-based DNAI1 protein analysis in cell, but none of them can accurately quantitate the amount of DNAI1 in tissues [21–23]. Ostrowski et al. tried various MS-based methods to analyze DNAI1 in the isolated cilia from cultured human bronchial epithelial (HBE) cells and found that DNAI1 can only be detected using 2-D or 1-D gel combined LC–MS/MS [21]. Recently, their group developed a label-free LC/MS^E^ method that can semi-quantitate over 400 cilla proteins in HBE cells including DNAI1 [22]. Marcotte et al. also detected DNAI1 protein when they used affinity-purification and mass-spectrometry (APMS) to identify protein interaction for inner and outer dynein arms of Xenopus animal caps [23].

In this work, a target proteomics method was developed and successfully qualified for human WT DNAI1 (hDNAI1) quantitation in human lung tissue. This method combined immunoprecipitation (IP) with a nanoLC-MS/MS platform to deliver a wide linear range, high sensitivity, and high accuracy for WT hDNAI1 quantitation in mouse and human lung tissues. Given the unique complexity of the lung matrix and the lack of accessibility to human DNAI1-free (i.e. PCD patient) lung tissues, a wild-type mouse lung was used as surrogate matrix to develop this method [24, 25]. Using this method, the endogenous concentrations of WT hDNAI1 in fourteen human lung tissues was successfully determined, demonstrating the great potential of this assay to expedite drug development for DNAI1-caused PCD therapy.

## Materials and methods

### Materials

Tissue Extraction Reagent I (TER-1), Dynabeads MyOne Streptavidin T1 beads (10 mg/mL) were purchased from Invitrogen (Carlsbad, CA). Pierce IP lysis buffer, Halt protease inhibitor cocktail 100x, MS-grade Trypsin/Lys-C protease mix, sequencing grade trifluoroacetic acid (TFA) were obtained from Thermo Scientific (Waltham MA). Minute Plasma membrane protein isolation and cell fractionation kit was from Invent Biotechnologies Inc (Plymouth, MN). Novagen Benzonase nuclease 10 kU (> 99%) was from Millipore (Burlington MA). 1,4-Dithiothreitol (DTT), Iodoacetamide (IAA), bovine serum albumin (BSA), 1 × phosphate buffered saline (PBS) were from Sigma (St Louis, MO). MS grade formic acid (FA), acetonitrile (ACN) and water were from Fisher (Waltham, MA). PBS containing 0.05% Tween 20 (PBST) 20 × was from Fluka (Buchs, Switzerland). RapiGest SF surfactant was ordered from Waters (Milford, MA). Recombinant human DNAI1 protein (His-tag, 0.15 µg/µL, ≥ 45%) was made by Genscript (Piscataway, NJ), and the protein concentration was calculated according to the purity provided by vendor. DNAI1 polyclonal antibodies were from Abnova (H00027019-D01P, Walnut, CA) and Invitrogen (PA5-54,526, Waltham, MA). Stable isotope labeled peptides (^13^C_6_^15^N_2_ labeled Lysine or ^13^C_6_^15^N_4_ labeled Arginine, > 95%) were synthesized by Biosynth (Gardner, MA). Fresh frozen wild-type CD-1 mouse lung tissues and wild-type human lung tissues were obtained from BioIVT (Hicksville, NY).

### Biotinylation of antibody

The antibody was biotinylated using water-soluble Sulfo ChromaLink Biotin (Vector Laboratories, Burlingame, CA) as described in the manufacturer’s instructions. Firstly, buffer exchange to amine free modification buffer was performed for the purified anti-human DNAI1 antibody solution. Subsequently, the antibody was diluted to 1 mg/mL concentration with amino free modification buffer. Then, the sulfo chromalink biotin solution was added to achieve an antibody/biotin molar ratio of 1:20. After incubation at room temperature for 2 h, the reaction was stopped by adding 5 µL of 0.1 M, pH 8.0 Tris–HCl buffer. Finally, a buffer exchange was conducted again to remove the unbound biotin, followed by resuspending antibody in modification buffer to a final concentration at 1 mg/mL.

### Tissue sample preparation

50 mg of mouse lung tissue was weighed and put into a 2 mL reinforced tube with three stainless steel beads (MP Biomedicals, Irvine CA). 450 µL of lysis buffer containing 1 × protease inhibitor cocktail and 0.1% (v/v) of benzonase nuclease was added. Then, the tissue was homogenized in the MP Fastprep-24^™^ 5G bead beating grinder and lysis system (Irvine CA) at a 6 m/s speed for 40 s for 2 cycles. The resultant homogenate was chilled on ice for 5 min, and then centrifuged at 16,000 × *g*, 4 ℃ for 20 min. The supernatant was carefully transferred to a KingFisher 96 deep well plate. Human lung tissue sample was prepared as described above, except that the homogenization was performed at a speed of 6 m/s for 30 s for 4 cycles.

### Preparation of standard and QC samples

Recombinant human DNAI1 protein was used to prepare standard and QC samples. Two sets of standard curves samples and 2-level QCs were made. To create an upper limit of quantitation (ULOQ) standard, 25 µL of 2 ng/µL DNAI1 protein solution was spiked into 50 mg tissue matrix, resulting in a concentration of 1000 pg/mg tissue. Other standard solutions were prepared by serially diluting this ULOQ solution by 3 times until limit of detection (LOD) standard achieved a 1.4 pg/mg tissue concentration. Two concentrations of QCs were prepared: 40 and 800 pg/mg tissue for low (QCL) and high (QCH) QC, respectively.

### Immunoprecipitation

Immunoprecipitation was carried out on a KingFisher Flex magnetic particle processor (Thermo Scientific, Waltham MA) using Dynabeads MyOne Streptavidin T1 magnetic beads (10 mg/mL, Invitrogen) and biotinylated anti-DNAI1 antibody. 450 μL/well of lung homogenate was dispensed into a KingFisher 96 deep-well plate. Next, 25 µL of 0.1 mg/mL biotinylated anti-human DNAI1 antibody solution was added to each well. The plate was then incubated in Thermomixer R (Eppendorf, Hamburg, Germany) at 4 ℃ with vortex at 600 rpm overnight. After incubation, 25 µL of 10 mg/mL streptavidin magnetic beads were added and then incubated at room temperature for 1 h with vigorous vortex at 1000 rpm. Subsequently, the magnetic beads were washed twice with 300 µL of 1 × PBST, followed by a wash with 300 µL of 1 ×× PBST/1 × PBS (1/2, v/v). Finally, the beads were resuspended in 100 µL of 0.1% RapiGest in 20 mM Tris–HCl buffer for subsequent on-beads digestion.

### On-beads digestion

The plate was initially heated at 95 ℃ with vortex at 1000 rpm for 5 min, and then allowed to cool down to room temperature. Subsequently, 10 µL of 50 mM DTT in 100 mM pH 8.0 Tris–HCl was added to each well. The plate was incubated with shaking at 1000 rpm at 55 ℃ for 45 min. After being cooled down to room temperature, each well was added with 10 µL of 150 mM IAA in 100 mM pH 8.0 Tris–HCl. The plate was incubated in the dark with shaking at 1000 rpm at room temperature for 30 min. Finally, 20 µL of 0.1 µg/µL Trypsin/LysC was added to each well. The plate was then incubated with shaking at 1000 rpm at 37 ℃ overnight for protein digestion.

After protein digestion was completed, the plate was placed on a magnetic rack for 5 min to separate the magnetic beads from sample solution. After bead separation, sample solution from each well was carefully transferred to a 1.5 mL protein Lobind centrifuge tube followed by adding 5 µL of 20% TFA to the sample tube individually. Then, the sample tubes were incubated at 37 ℃ for 40 min, and centrifuged at 20,000 × *g*, 4 ℃ for 20 min. The supernatant was transferred to a new tube and a mixture of heavy peptides was spiked into each sample as internal standards to achieve 2 fmol/µL of final concentration.

Peptide cleanup was performed on a SOLAµ SPE plate. SPE cartridge was activated first with 100% ACN, and then equilibrated with 0.1% FA. A sample was slowly loaded onto the SPE cartridge, followed by washing the cartridge with 0.1% FA for three times. Next, peptides were eluted slowly with 0.1% FA in 70% ACN. The eluate containing purified peptides was dried down on a SpeedVac system, and the resultant peptides were reconstituted in 10 µL of 0.1% FA.

### NanoLC-MS/MS

Tryptic peptide sample analysis was conducted on an EASY-nLC 1200 system coupled with Q Exactive HF-X mass spectrometer (Thermo Scientific, Waltham MA). LC separation was performed on a Thermo PepMap C18, 1.8 µm, 75 µm ID × 150 mm column (Thermo Scientific, Waltham MA) at a flow rate of 300 nL/min with column temperature set to 45 ℃. 0.1% FA in DI water and 0.1% FA in 90% ACN were used as mobile phase A and B, respectively. The gradient was set as 2% B (0 min)-40% B (30 min)- 95% B (35 min)-95% B (40 min)-2% B (45 min)-2% B (50 min). MS data were collected by parallel reaction monitoring (PRM) acquisition in the positive ion mode, with MS2 resolution of 45,000, AGC target at 3e6, Maximum IT of 120 ms, isolation window at 1.0 m/z and (N)CE of 30. The precursors from surrogate peptide AHIFDLAINK and AHIFDLAINK^ (^13^C_6_^15^N_2_ labeled Lysine) were monitored at *m/z* 571.3218 and 575.3289 representing light and heavy peptide, respectively.

### Data analysis

The MS data generated in data-dependent acquisition mode was analyzed using Biopharma Finder software (Version 3.0, Thermo Fisher) and Proteome Discoverer (Version 2.4, Thermo Fisher). Skyline software (MacCross lab) was utilized to process MS data for targeted proteomics method development. Peak area of each surrogate peptide ion was generated by Xcalibur software (Version 4.0, Thermo Fisher). All peak integrations underwent visual inspection to ensure acceptable integration quality. The quantitation of peptide AHIFDLAINK was performed using transitions 571.3218 (Q1) → 933.5404 (Q3) and 575.3289 (Q1) → 941.5546 (Q3) for light and heavy form, respectively. Peak area ratio of analyte to IS and their nominal concentrations were used to generate the standard curve. A 1/X-weighted linear regression was applied to improve the curve fitting.

## Results and discussion

### Method development

**Fig. 1:**
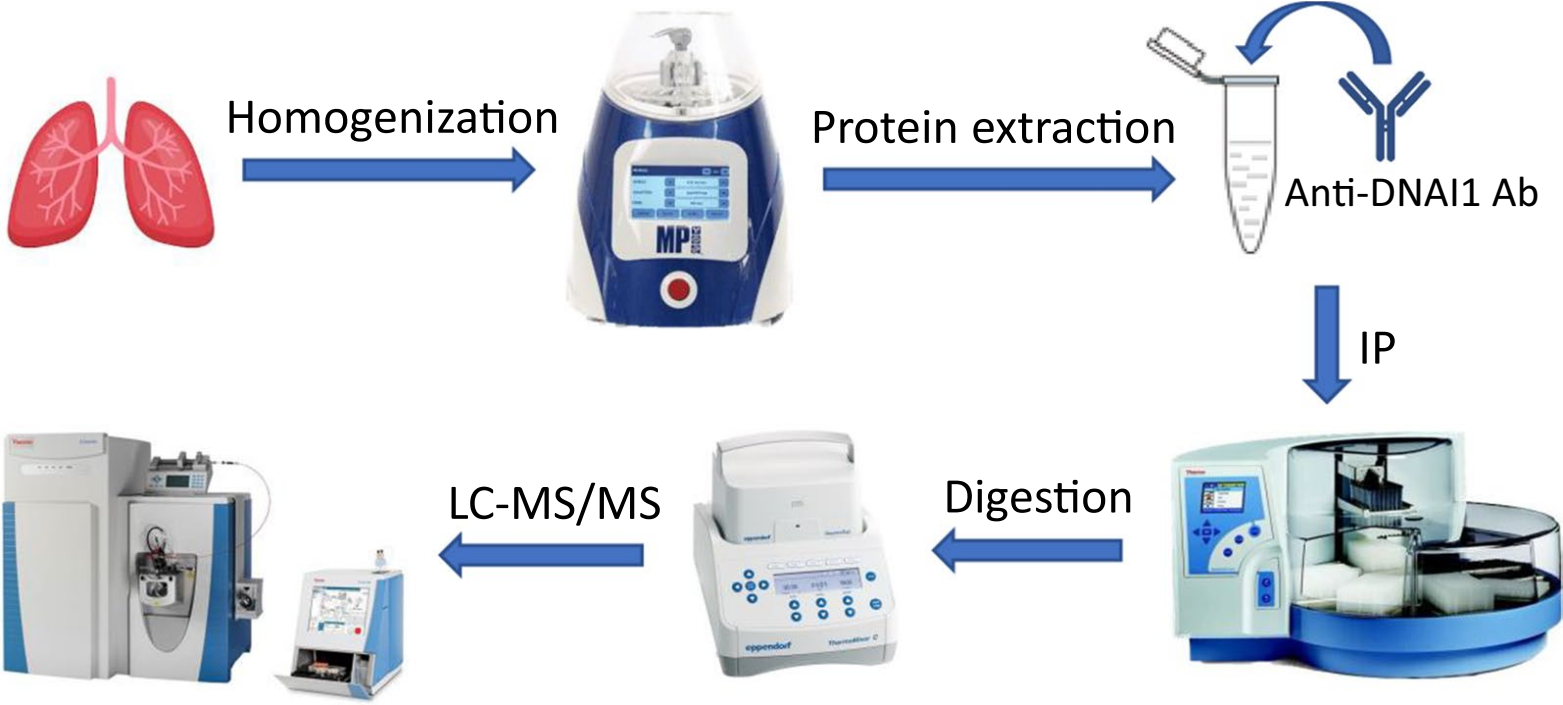
Workflow of IP-nanoLC-MS/MS analysis

This work aimed to develop and qualify a LC–MS method that can accurately quantitate the absolute amount of extremely low abundant DNAI1 protein in lung tissue. To achieve this goal, a strategy that combines an IP approach with a nanoLC-PRM-MS technique was employed. Figure 1 showed the workflow of this new method. Briefly, lung tissue is homogenized, and proteins are extracted, followed by IP using anti-DNAI1 antibody. The enriched protein is then digested on beads with trypsin/LysC and resultant tryptic peptides are analyzed by nanoLC-PRM-MS.

Protein identity was confirmed by Biopharma Finder using data-dependent-acquisition (DDA) MS data against human DNAI1 sequence (UniProtKB Q9UI46). Surrogate peptide selection was conducted using peptide mapping data generated from in-solution trypsin/LysC digestion of recombinant human DNAI1 protein (Additional file 1: Fig. S1). Top seven unique human DNAI1 peptides were selected as surrogate candidates for hDNAI1 quantitation (Additional file 2: Table S1) based on the “golden standard” of peptide selection criteria for targeted proteomics, including the uniqueness of the peptide, mis-cleavage, MS signal, peptide length, hydrophobicity, post translational modification, etc. [26–28]. Signal intensities of all precursor *m/z* of each surrogate peptide candidate were compared and its collision energy (CE) was also optimized through using each peptide’s corresponding stable isotope labeled counterpart. All the parameters were refined by performing a LC-PRM run using recombinant DNAI1 protein digests spiked with IS peptides and checking the retention time and MS response of each peptide candidate.

### Antibody screening

To determine the feasibility of the IP-MS method for DNAI1 protein quantitation, the protein extract from mouse lung tissue homogenate with and without spiked recombinant hDNAI1 protein was tested as positive and negative control samples. The data showed that all the seven hDNAI1 peptide candidates were detected in the hDNAI1 spiked-in samples after IP, while no hDNAI1 peptide was detected in mouse lung negative control sample. Additional file 1: Fig. S2 shows chromatograms of seven peptides candidates in spiked mouse lung tissue. This result demonstrated the feasibility and specificity of IP-MS assay for hDNAI1 protein quantitation.

Five antibodies were tested for IP. Based on the initial result, two antibodies from Abnova and Invitrogen, respectively, were selected for further investigation due to their higher specificity and binding affinity in comparison to others (data not shown). Since our previous research have proven that streptavidin beads-biotinylated antibody IP approach can deliver higher specificity than Protein G-antibody IP approach, and that the on-beads digestion can yield higher peptide recovery than off-beads digestion [29], the biotinylated anti-hDNAI1 antibody and streptavidin T1 magnetic beads was used in IP followed by on-beads digestion.

**Fig. 2:**
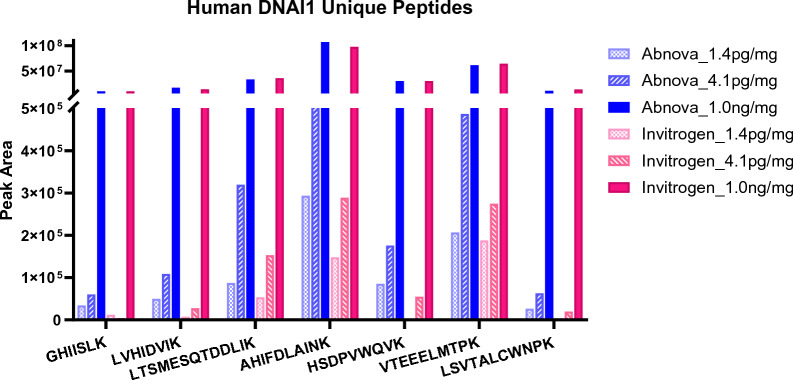
Comparison of peak areas of seven hDNAI1 unique peptides that resulted from IP test of Abnova antibody *vs* Invitrogen antibody. Concentrations of spiked DNAI1 in pooled wild-type CD-1 mouse lung tissue were 1000, 4.1 and 1.4 pg/mg tissue, respectively

Further investigation on Abnova and Invitrogen antibodies was conducted with DNAI1 protein at 1000, 4.1 and 1.4 pg/mg tissue concentrations that can cover a linear range of standard curve for hDNAI1 quantitation including both the ULOQ and LLOQ. Figure 2 shows a comparison of peak areas of seven hDNAI1 unique peptides that resulted from IP testing Abnova and Invitrogen antibodies, respectively. The data show that when hDNAI1 concentration is 1000 pg/mg tissue, peak areas of these seven peptides are comparable, indicating that these two antibodies performed similarly in the IP of hDNAI1 protein from lung tissue for high concentration of DNAI1. However, at low hDNAI1 concentrations, Abnova antibody demonstrated much higher efficiency than Invitrogen’s. When the tested hDNAI1 concentration was 4.1 pg/mg tissue, the peak areas of seven peptides that resulted from Abnova antibody IP were at least twofold higher than that from Invitrogen’s. At an even lower concentration of 1.4 pg/mg tissue, four peptides, GHIISLK, LVHIDVIK, HSDPVWQVK and LSVTALCWNPK, were undetectable in the Invitrogen antibody IP but remained detectable with Abnova’s. This compelling result led to the selection of Abnova antibody in the DNAI1 IP quantification method.

### Lysis buffer selection

Since lysis buffer was also used as the loading buffer for immunoprecipitation and can significantly impact the IP efficiency, three lysis buffers were assessed to test hDNAI1 protein recovery: Pierce IP lysis buffer (Pierce IP, Invitrogen, Carlsbad, CA), Tissue extraction reagent I (TER-1, Thermo Scientific, Waltham, MA), and Buffer A from the Minute plasma membrane protein isolation and cell fractionation kit (Buffer A, Invent Biotechnologies Inc, Plymouth, MN). The assessment was carried out by using mouse lung tissues spiked with recombinant hDNAI1 as test samples. The lysis buffer was introduced to mouse tissue at a tissue/lysis buffer ratio of 1:9, followed by the tissue homogenization and protein extraction. Two replicates were tested in each lysis buffer condition.

**Fig. 3:**
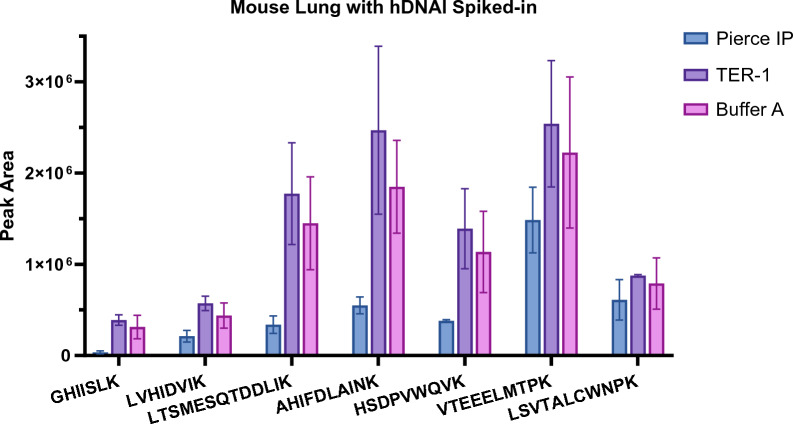
Comparison of peak areas of hDNAI1 peptides that resulted from IP tests of hDNAI1 spiked pooled wild-type CD-1 mouse lung tissue using three lysis buffers: Pierce IP, TER-1, and Buffer A, respectively

Figure 3 shows the comparison of peak areas of hDNAI1 peptides resulted from IP tests using the three lysis buffers. TER-1 lysis buffer showed the highest hDNAI1 recovery, which was approximately 1.2-fold higher than that of Buffer A, and up to 11 folds higher that of Pierce IP. This result suggested that TER-1 lysis buffer can lead to an improved hDNAI1 recovery during IP and subsequently resulted in a higher method sensitivity. BCA assay showed the protein yield after IP for mouse lung homogenate to be approximately 5.9 µg/µL for Pierce IP, 8.2 µg/µL for TER-1, and 1.6 µg/µL for Buffer A. Protein ID analysis of tested sample after IP identified 2188 for Pierce IP buffer, 1830 for TER-1, and 1789 for Buffer A as IP loading buffer, respectively. These results confirmed that TER-1 not only enables higher protein binding for hDNAI1 to antibody, but also reduces the binding of non-specific proteins, ultimately enhancing the IP efficiency (Additional file 1: Fig. S3).

**Fig. 4:**
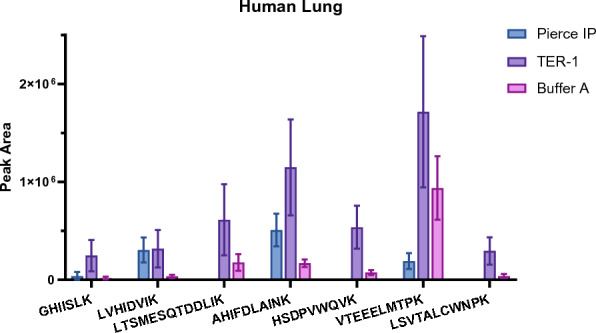
Comparison of peak areas of hDNAI1 peptides that resulted from IP tests of wild-type human lung tissue using three lysis buffers: Pierce IP, TER-1, and Buffer A, respectively

The IP efficiency of lysis buffers for hDNAI1 extraction was also investigated in human lung tissue. Two replicates, each containing a 50 mg section of wild-type (WT) normal human lung tissue, were homogenized with the three lysis buffer candidates, followed by IP-MS analysis, respectively. Consistent with test results in mouse lung tissue, TER-1 lysis buffer demonstrated the highest protein yields for IP and the highest sensitivity towards hDNAI1 protein detection on MS (Fig. 4). This result confirmed the TER-1 is the most favorable buffer for IP among those evaluated.

### Method qualification

**Fig. 5:**
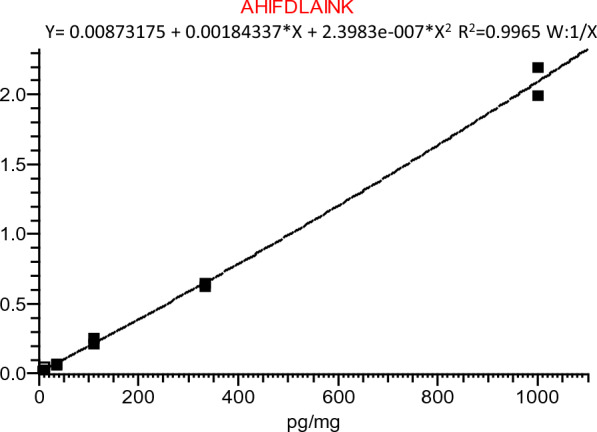
Standard curves of peak area ratio of light to heavy peptide AHIFDLAINK *vs* different spiked hDNAI1 protein concentrations in pooled wild-type CD-1 mouse lung tissue. hDNAI1 concentrations were 1.4, 4.1, 12, 37, 111, 333 and 1000 pg/mg tissue for standard 1 to standard 7, respectively

After optimization, peptide AHIFDLAINK (AA 607–616) was finally selected for quantitating hDNAI1 protein due to its best performance in hDNAI1 spiked in mouse matrix and human lung tissues. Although peptide VTEEELMTPK shows higher MS response, it was not selected due to the potential oxidation of Methionine. The IP-MS assay was then qualified in mouse lung tissue through assessing the linearity, sensitivity, accuracy, and precision of standards and quality controls (QCs). A good linearity of the peak area ratio of target peptide to its isotope-labeled internal standard was observed. The linear range spanned from 4.1 to 1000 pg hDNAI1 per mg tissue. Figure 5 showed a standard curve with R^2^ value of 0.996. The limit of detection (LOD) was 1.4 pg/mg tissue with a S/N greater than 3. The lower limit of quantitation (LLOQ) for hDNAI1 in mouse lung tissue was 4.1 pg/mg tissue (Additional file 1: Fig. S4). The back-calculated concentration of calibration standards from 7 LC–MS runs from 5 different batches shows good accuracy of this assay, with % Bias ≤ 10% for all standards, and good precision with CV% < 25% for inter-batch runs. And two-level concentrations of QCs were also examined. QCs of 4 different LC–MS runs from 3 batches shows good accuracy and precision, with inter-batch QC error% within ± 15% and CV% no more than 15% for QC low (Additional file 2: Table S2).

### Human lung tissue analysis

The successfully qualified assay was subsequently employed in quantitating endogenous hDNAI1 protein in wild-type human lung tissue. Using mouse lung tissue as surrogate matrix, the endogenous level of human DNAI1 was successfully quantified in fourteen WT human lung tissue samples, ranging from 4.3 to 45 pg/mg tissue (details provided in Table 1). The variability of measured hDNAI1 concentrations in WT human lung tissues could be caused by the variable samples sourced from different human lung sections where DNAI1-containing epithelial cells are not distributed evenly or the different expression levels of hDNAI1 in individuals. The successful quantitation of low-abundance DNAI1 protein in human lung tissue samples represents a notable achievement of this new IP-MS assay. Its ability to accurately measure low level of DNAI1 in human lung tissue opens new avenues for studying the role of this protein in various biological processes and disease conditions. Overall, this IP-MS assay offers a valuable tool for investigating the complex biology of low abundant hDNAI1 protein, can have broad implications for various biomedical and preclinical applications.

**Table 1:** IP-MS analysis for wild-type human lung

Sample	**Lot #**	Calculated concentration (pg/mg tissue)*
Human lung 01	MHU-P-051915	27.1
Human lung 02	MHU-P-082719	4.37
Human lung 03	MHU-P-082919	14.4
Human lung 04	P1245-Tn8	14.3
Human lung 05	P1060-Tn8	5.82
Human lung 06	P1079-Tn8	9.24
Human lung 07	P1140-Tn10	4.40
Human lung 08	P1053-Tn7	44.7
Human lung 09	K30-Tn7	7.15
Human lung 10	R64-Tn8	12.4
Human lung 11	P1096-Tn7	5.03
Human lung 12	P1574-Tn10	45.6
Human lung 13	P1034-Tn9	19.6
Human lung 14	P1113-Tn10	31.9

^*^Calculated concentration (pg/mg tissue) of hDNAI1 was determined based on the standard curve for the peptide AHIFDLAINK from the hDNAI1 protein in tissue matrices

## Conclusion

A highly sensitive and selective LC–MS assay for quantitating hDNAI1 protein in human lung tissue matrix was developed through combining a protein immunoprecipitation strategy with a nanoLC-MS/MS technique. Furthermore, the assay was successfully qualified. The IP conditions, including specific antibody and loading buffer were optimized to achieve the desired sensitivity. This qualified assay shows a linear dynamic range with up to 3 orders of magnitude for hDNAI1 quantitation in lung tissue matrix. The LLOQ was 4.1 pg hDNAI1 per mg tissue. The successful quantitation of endogenous hDNAI1 protein in WT human lung tissues indicated the great potential of this new assay to support preclinical/clinical development of mRNA-based therapeutics for PCD.

### Supplementary Information

**Additional file 1: Fig. S1.** Peptide mapping for recombinant human DNAI1 protein with trypsin/LysC digestion. **Fig. S2.** IP-MS analysis for human DNAI1 spiking in mouse lung matrix (**A**) and mouse lung matrix only (**B**). 1, hDNAI1 peptide AHIFDLAINK, 2, hDNAI1 peptide HSDPVWQVK. **Fig. S3.** Venn diagrams of protein ID numbers after IP-MS using Pierce IP, TER-1, Buffer A lysis buffer as IP loading buffer. **Fig. S4.** LOD (A) and LLOQ (B) of IP-MS assay for human DNAI1 in mouse lung matrix. 1, endogenous peptide AHIFDLAINK; 2, isotope labeled peptide AHIFDLAINK^.

**Additional file 2: Table S1.** Human DNAI1 peptide candidates. **Table S2. **Accuracy and precision of standards and QC samples. **Table S3. **Summary of protein ID numbers after IP-MS using Pierce IP,TER-1, Buffer A lysis buffer as IP loading buffer.

## Data Availability

All data generated and analyzed during this study are included in the published article and its Additional files.

## Abbreviations

DNAI1: Dynein axonemal intermediate chain 1 protein
PCD: Primary ciliary dyskinesia
MS: Mass spectrometry
LC–MS/MS: Liquid chromatography-tandem mass spectrometry
IP: Immunoprecipitation
PRM: Parallel reaction monitoring
WT: Wild type
hDNAI1: Human DNAI1
mRNA: Messenger RNA
TER-1: Tissue Extraction Reagent I
DDA: Data dependent acquisition
CE: Collision energy
QC: Quality control
LOD: Limit of detection
LLOQ: Lower limit of quantitation
CV: Coefficient of variation
S/N: Signal to noise ratio
