# Supplementary material for: Absolute quantitation of human wild-type DNAI1 protein in lung tissue using a nanoLC-PRM-MS-based targeted proteomics approach coupled with immunoprecipitation
Source: Clin Proteomics. 2024 Feb 4;21:8. doi: 10.1186/s12014-024-09453-0 (PMC10840268; doi:10.1186/s12014-024-09453-0)
Supplement: Supplementary file 1 — Additional file 1: Fig. S1. Peptide mapping for recombinant human DNAI1 protein with trypsin/LysC digestion. Fig. S2. IP-MS analysis for human DNAI1 spiking in mouse lung matrix (A) and mouse lung matrix only (B). 1, hDNAI1 peptide AHIFDLAINK, 2, hDNAI1 peptide HSDPVWQVK. Fig. S3. Venn diagrams of protein ID numbers after IP-MS using Pierce IP, TER-1, Buffer A lysis buffer as IP loading buffer. Fig. S4. LOD (A) and LLOQ (B) of IP-MS assay for human DNAI1 in mouse lung matrix. 1, endogenous peptide AHIFDLAINK; 2, isotope labeled peptide AHIFDLAINK^. [file 12014_2024_9453_MOESM1_ESM.pptx]

## Slide 1
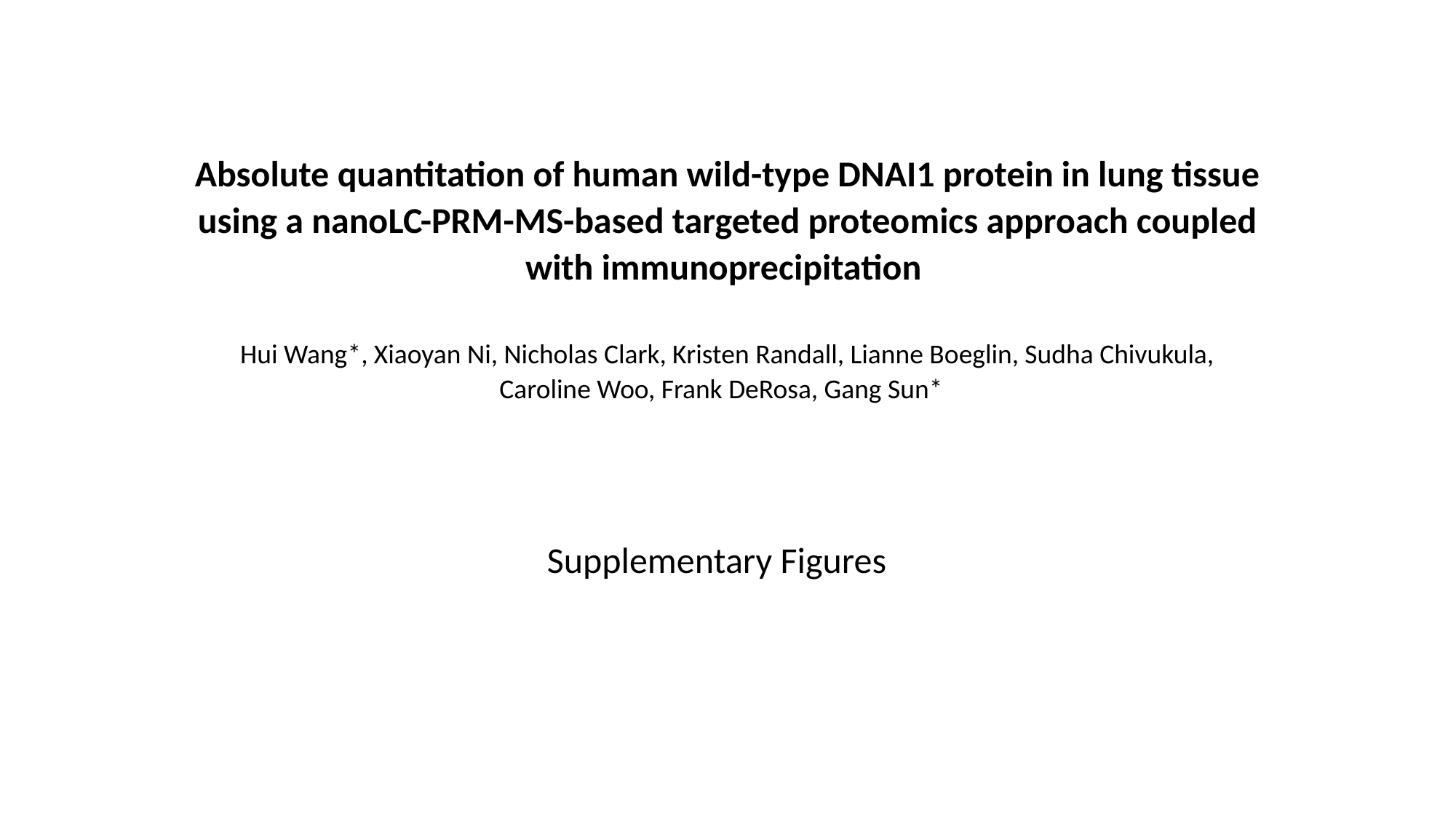

Absolute quantitation of human wild-type DNAI1 protein in lung tissue using a nanoLC-PRM-MS-based targeted proteomics approach coupled with immunoprecipitation
 
Hui Wang*, Xiaoyan Ni, Nicholas Clark, Kristen Randall, Lianne Boeglin, Sudha Chivukula, Caroline Woo, Frank DeRosa, Gang Sun*
Supplementary Figures

## Slide 2
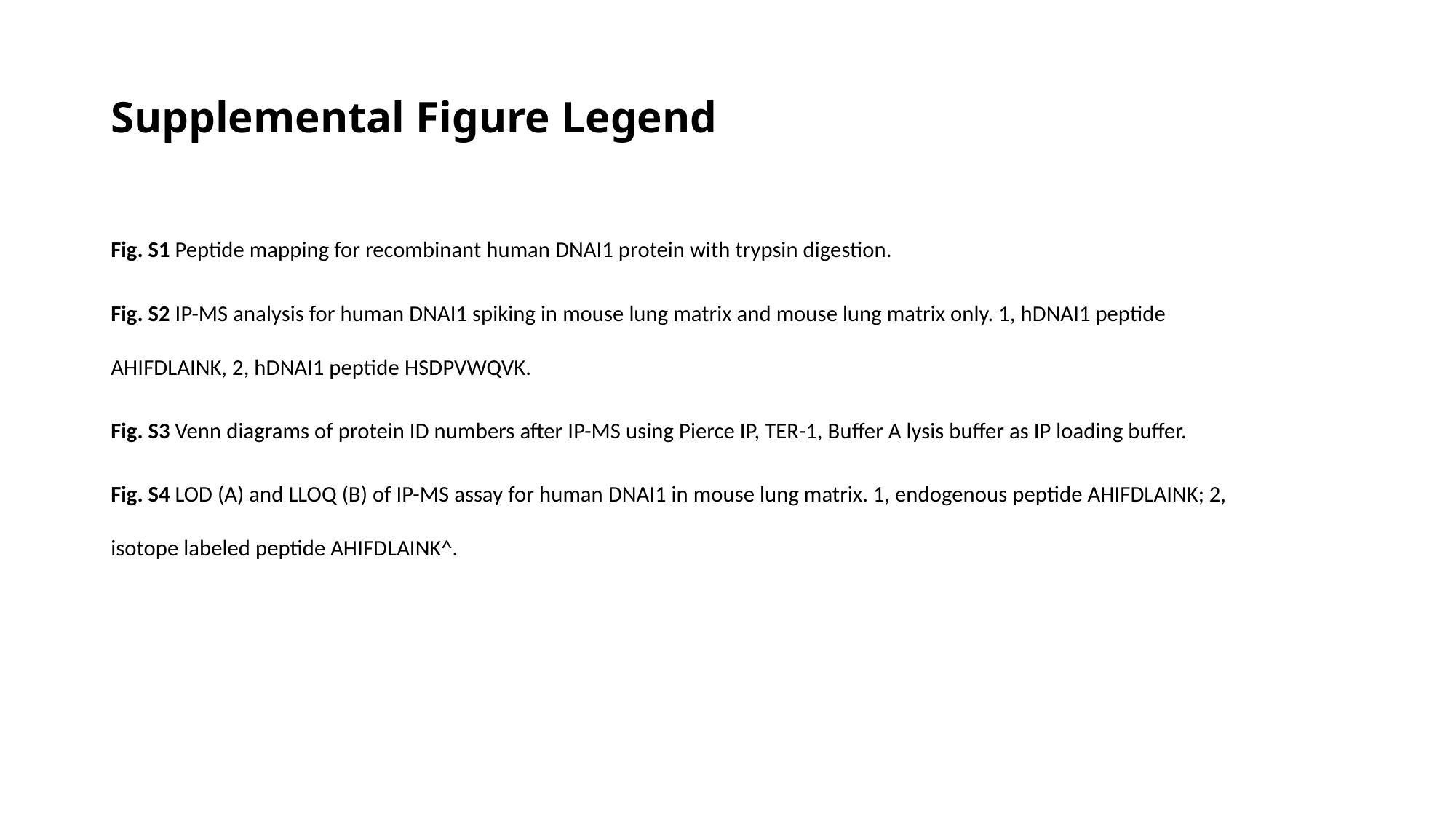

# Supplemental Figure Legend
Fig. S1 Peptide mapping for recombinant human DNAI1 protein with trypsin digestion.
Fig. S2 IP-MS analysis for human DNAI1 spiking in mouse lung matrix and mouse lung matrix only. 1, hDNAI1 peptide AHIFDLAINK, 2, hDNAI1 peptide HSDPVWQVK.
Fig. S3 Venn diagrams of protein ID numbers after IP-MS using Pierce IP, TER-1, Buffer A lysis buffer as IP loading buffer.
Fig. S4 LOD (A) and LLOQ (B) of IP-MS assay for human DNAI1 in mouse lung matrix. 1, endogenous peptide AHIFDLAINK; 2, isotope labeled peptide AHIFDLAINK^.

## Slide 3
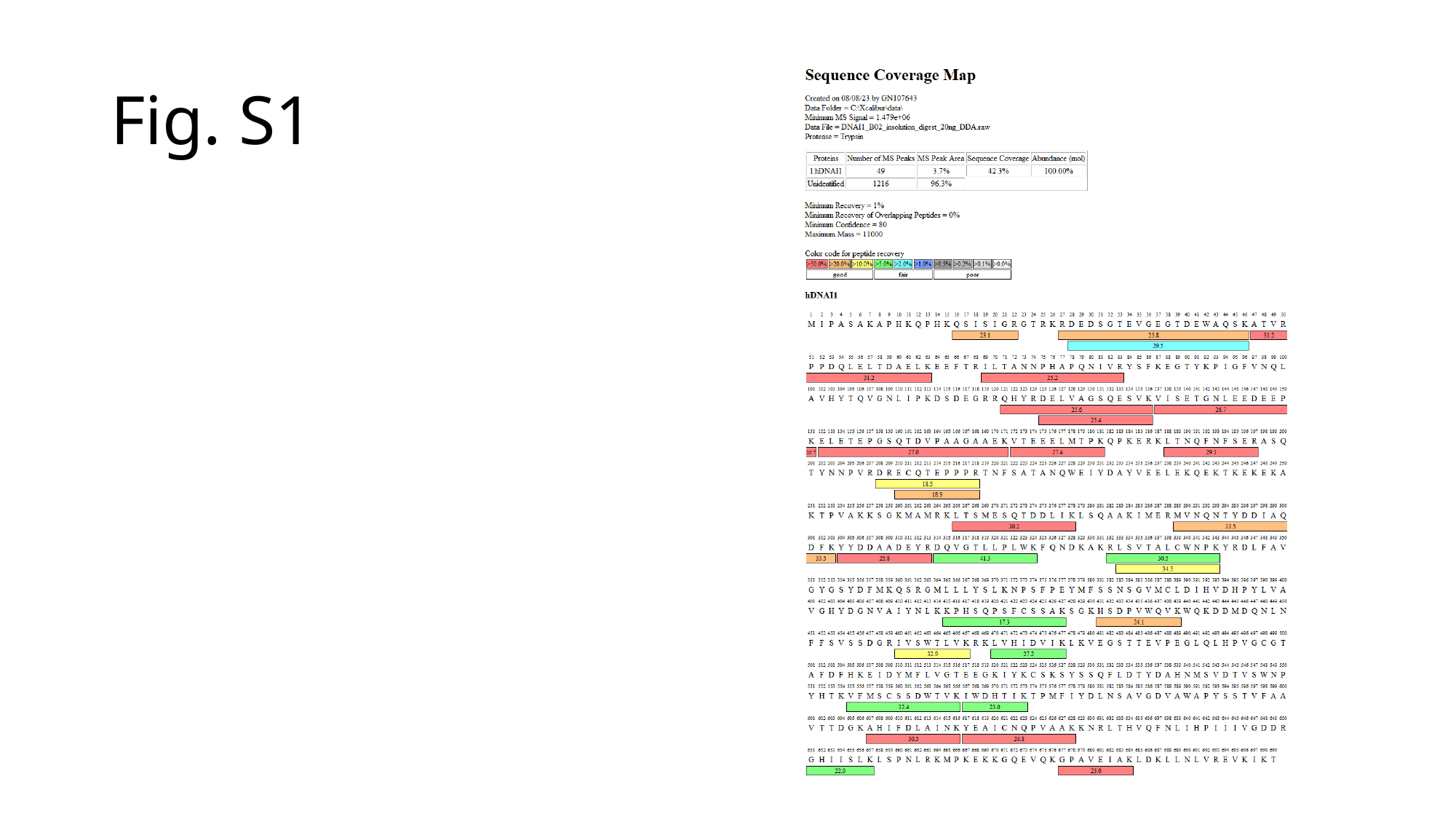

# Fig. S1

## Slide 4
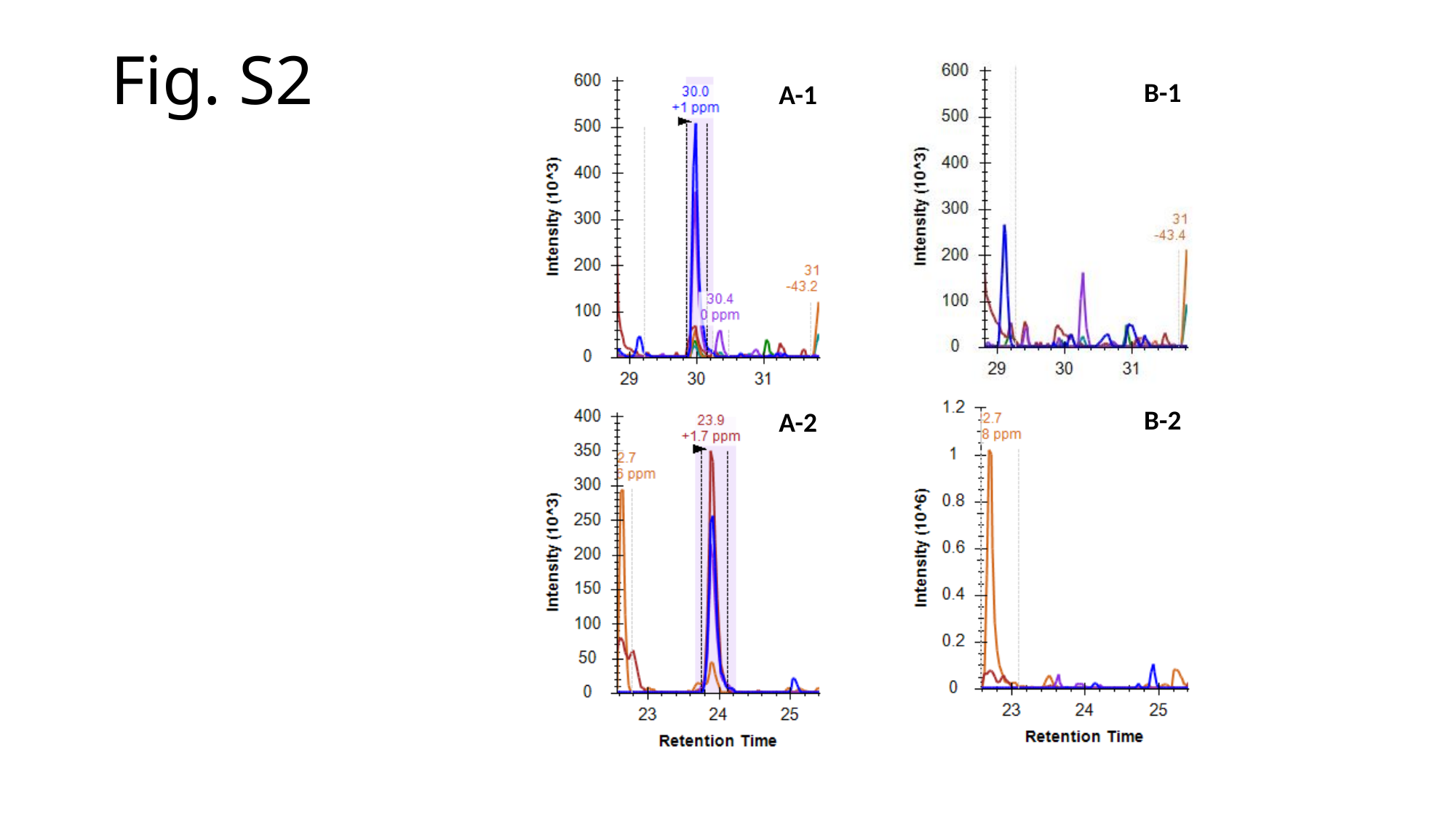

# Fig. S2
B-1
B-2
A-1
A-2

## Slide 5
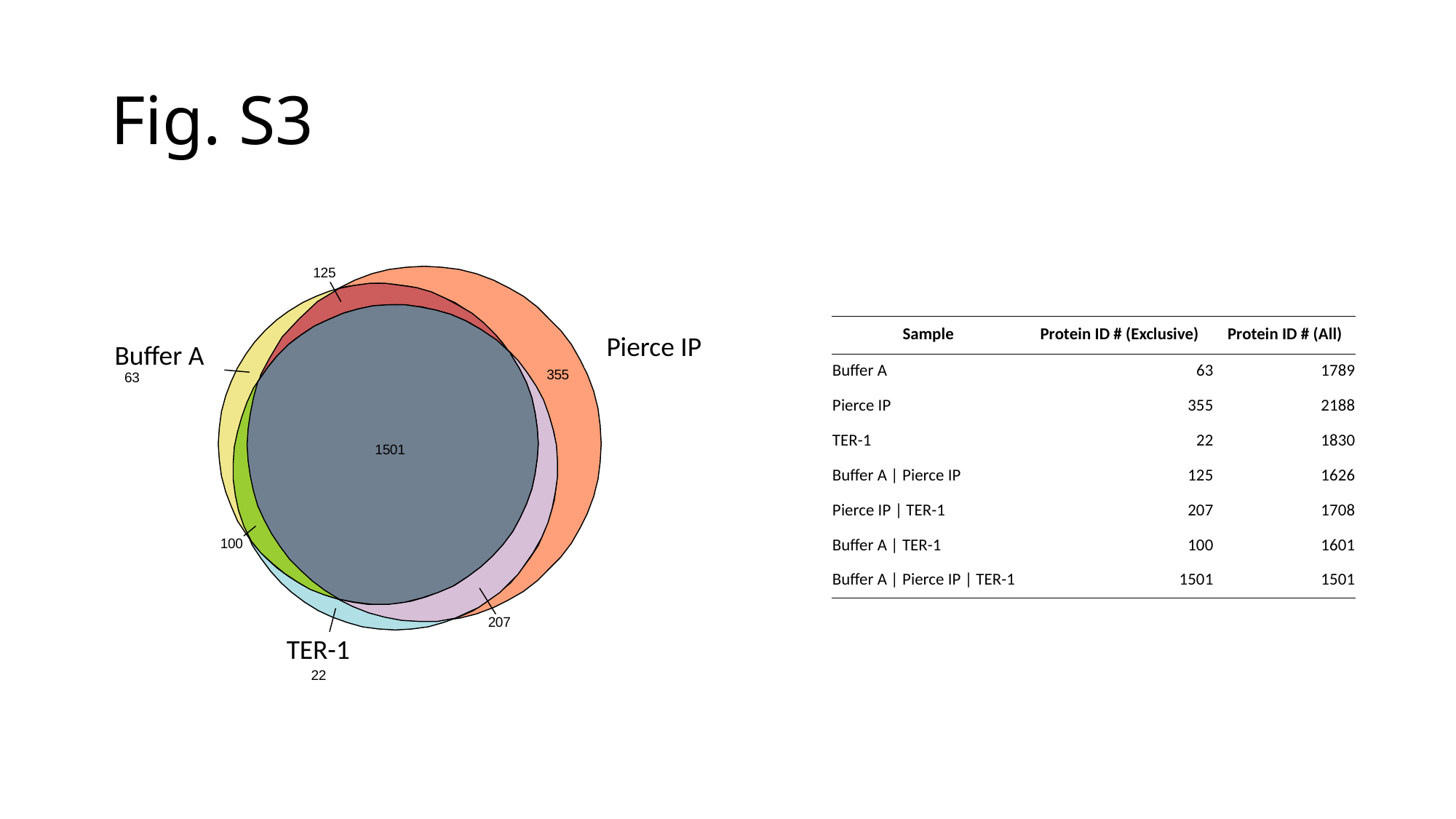

# Fig. S3
| Sample | Protein ID # (Exclusive) | Protein ID # (All) |
| --- | --- | --- |
| Buffer A | 63 | 1789 |
| Pierce IP | 355 | 2188 |
| TER-1 | 22 | 1830 |
| Buffer A | Pierce IP | 125 | 1626 |
| Pierce IP | TER-1 | 207 | 1708 |
| Buffer A | TER-1 | 100 | 1601 |
| Buffer A | Pierce IP | TER-1 | 1501 | 1501 |
Pierce IP
Buffer A
TER-1

## Slide 6
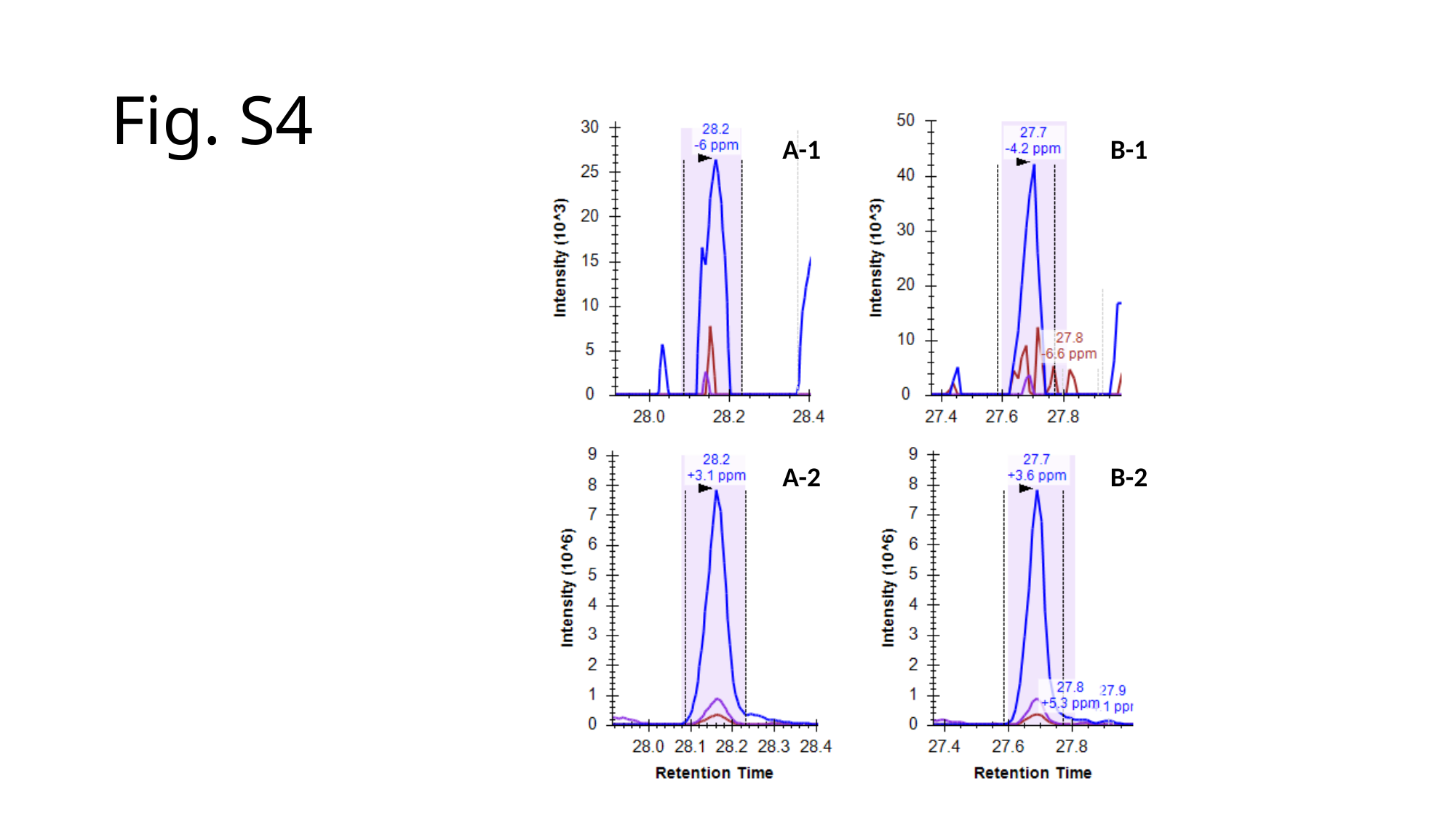

# Fig. S4
A-1
A-2
B-1
B-2
